# Quantifying Temperature Dependence of Pu(IV) Absorbance Spectra for Advanced Online Monitoring of Nuclear Processes

**DOI:** 10.1021/acs.analchem.6c01635

**Published:** 2026-06-20

**Authors:** Sara E. Gilson, Cannon J. Giglio, Hunter B. Andrews, Kristian G. Myhre, Luke R. Sadergaski

**Affiliations:** Radioisotope Science and Technology Division, 6146 Oak Ridge National Laboratory, 1 Bethel Valley Road, Oak Ridge, Tennessee 37830, United States

## Abstract

This article presents a systematic study of Pu­(IV) absorbance spectral features as a function of temperature to develop an understanding of this parameter’s effect on chemometric models that can be used as online monitoring tools to support nuclear processing. The descriptive and predictive models that provide real-time feedback of these processes are usually constructed with data collected in conditions typical of a laboratory environment, which can differ drastically from a processing environment. To assess the impact of temperature on Pu­(IV) absorbance spectra, 11 samples of Pu­(IV) were synthesized with varying HNO_3_ concentrations ranging from 0.6 to 9.5 M and heated between 15 and 45 °C. Ultraviolet (UV)–visible (vis)–near-infrared (NIR) absorption spectra collected at different HNO_3_ concentrations and temperatures revealed that features associated with Pu­(IV) are sensitive to temperature at all HNO_3_ concentrations and that changes in features depend on HNO_3_ concentration. The contributions of temperature and HNO_3_ concentration to variation in Pu­(IV) spectral features were evaluated using the principal component analysis of spectra that were baseline-corrected with an asymmetric least-squares method. Furthermore, predictive modeling for HNO_3_ concentration with partial least-squares regression of UV–vis–NIR spectra highlighted the importance of accounting for temperature in the calibration set to optimize model performance. This methodology constitutes a new, systematic approach to account for the effect of temperature on the absorption spectra of metal ions and is useful for process monitoring applications in many industries.

## Graphical abstract

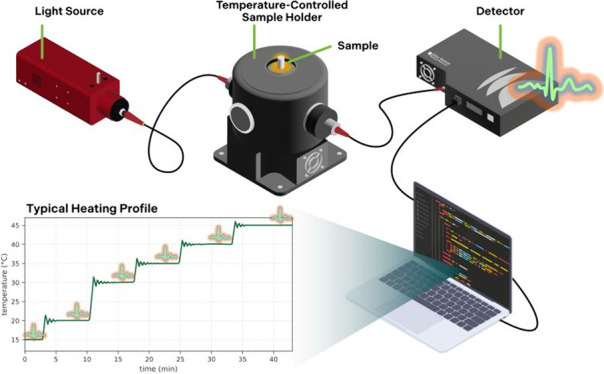

## Introduction

Nuclear processing benefits from online monitoring tools and their continued development for real-time, in situ feedback.^1,2^ However, tools such as descriptive and predictive models are usually built using data collected in conditions typical of a laboratory setting, which can differ drastically from a processing environment.^3–5^ Such discrepancies between these two settings can affect the chemistry and sensor signals used for online monitoring. This issue is especially problematic for systems monitoring, for example, Pu, whose absorbance spectra are also highly sensitive to other factors, such as redox behavior and solution acidity.^6–10^

Although the absorbance spectrum of Pu­(IV) is known to change with HNO_3_ concentration, the effect of temperature on its spectrum is not well-studied.^11–13^ Harsh radiological environments for nuclear processing are usually multiple degrees Celsius higher in temperature than ambient laboratory conditions. Temperature is a key variable that affects Pu process chemistry; it can cause changes in Pu oxidation state chemistry as well as shifts and changes in spectral features.^1,14^ As temperature changes, the energy distribution among electronic states shifts, which leads to changes in the intensity and position of spectral bands.^15–17^ Previous work determined that applying heat to Pu in 3 M HNO_3_ oxidized Pu­(IV) to Pu­(VI).^14^ However, to the best of our knowledge, no systematic study of the effect of temperature on Pu­(IV) absorbance spectra across multiple HNO_3_ concentrations has been reported in the literature.^7,14,18^

Addressing the lack in understanding of the contribution of temperature to Pu­(IV) spectral variance can help optimize the performance of partial least-squares regression (PLSR) models.^19^ Strong performance of these tools is crucial for ensuring efficient and safe Pu processing.^20^ Accounting for temperature-driven changes in Pu­(IV) spectral features when building models for online monitoring may lead to more accurate descriptive and predictive performance, such as HNO_3_ quantification.

This study systematically investigated the effect of temperature on Pu­(IV) absorbance spectral features over a wide range of HNO_3_ concentrations, which, to the best of our knowledge, has not been reported previously. To differentiate and quantify contributions of HNO_3_ concentration and temperature to variance in Pu­(IV) spectra, principal component analysis (PCA) was applied to this unique data set. Finally, the effect of temperature on PLSR model performance was assessed by tuning the temperature of the data included in the calibration set. This methodology constitutes a new approach for quantifying the effect of temperature on the absorption spectra of metal ions.

## Experimental Section

### Caution

The isotope ^240^Pu (*t*_1/2_ = 6564 years) is an α-emitting radioisotope and presents a significant health hazard to workers. To mitigate this risk, all work was executed in facilities equipped to handle this radioisotope. Sample synthesis, manipulations, and spectroscopic measurements were carried out in a negative-pressure glovebox.

### Chemicals

The stock solution of ^240^Pu was obtained in-house at the US Department of Energy’s Oak Ridge National Laboratory. To purify the Pu, the stock solution was acidified to 7.5 M HNO_3_, loaded onto a column with preconditioned MP-1 anion exchange resin, and washed with four bed volumes of 7.5 M HNO_3_ and then two bed volumes of 3 M HNO_3_. Finally, 0.5 M HNO_3_ was used to elute the Pu from the column. The concentration of the purified Pu stock solution was determined through alpha spectroscopy (Canberra Alpha Spectrometer model 7401). This stock solution was used to synthesize all Pu samples in this study. HNO_3_ (Merck, 65% for analysis) and Milli-Q purity H_2_O were used to prepare all samples.

### Sample Preparation

A set of samples with the same Pu concentration and different HNO_3_ concentrations was synthesized. The Pu stock solution was diluted with known volumes of concentrated HNO_3_ and Milli-Q purity H_2_O to achieve the desired sample concentrations. Sample compositions are given in Table 1. Select samples were analyzed for Pu concentration and [H^+^] by alpha spectroscopy and potentiometric titrations, respectively. Due to the high specific activity of ^240^Pu, it was not possible to analyze all sample compositions.

**Table 1:** Pu Sample Target Compositions and Temperatures of Spectral Measurements

**sample**	**[Pu] (M)**	**[HNO**_**3**_ **] (M)**	**temperatures (°C)**
Pu S0^a^	0.028	0.6	15, 20, 30, 35, 40, 45
Pu S1	0.028	1.0	15, 20, 30, 35, 40, 45
Pu S2	0.022	1.3	15, 20, 30, 35, 40, 45
Pu S3^a^	0.028	2.3	15, 20, 30, 35, 40, 45
Pu S4	0.028	3.3	15, 20, 30, 35, 40, 45
Pu S5	0.028	4.3	15, 20, 30, 35, 40, 45
Pu S6	0.028	5.0	15, 20, 30, 35, 40, 45
Pu S7^a^	0.028	6.0	15, 20, 30, 35, 40, 45
Pu S8	0.028	7.0	15, 20, 30, 35, 40, 45
Pu S9	0.028	8.0	15, 20, 30, 35, 40, 45
Pu S10^a^	0.028	9.5	15, 20, 30, 35, 40, 45

a Underwent α-spectroscopy and potentiometric titration to confirm [H^+^]

### Temperature-Controlled Absorbance Spectroscopy

An Avantes CUV-UV/vis-TC temperature-controlled cuvette holder equipped with a Peltier-cooled device was used to control temperature, as described previously.^21^ Spectral measurements were conducted using a quartz cuvette with a 1.75 mm path length. All measurements were referenced against Milli-Q purity H_2_O at 15 °C. For each measurement, 400 μL of sample was pipetted into the cuvette, and spectra were assessed for nitrite formation in the UV region as well as other anomalous signals before temperature adjustment.^22^ Nitrite formation was not detected in any of the samples.

UV–vis spectra were collected with a QEPro spectrophotometer (Ocean Insight) using a stabilized W–halogen lamp (ThorLabs) as the light source. UV–vis spectra were collected from 320 to 1115 nm every 0.80 nm. Spectra were collected in triplicate at each target temperature after the sample had equilibrated for approximately 8 min and the thermocouple in the cuvette holder indicated temperature stabilization. The target temperatures are given in Table 1, and a representative sample heating protocol for spectra collection is given in Figure S1. Temperature measurements typically lasted approximately 45 min. Temperature measurements of samples were collected as soon as was feasible after being synthesized, typically a few days to 1 week. Spectra were collected 12 h after synthesis (to allow for equilibration) as well as right before temperature experiments and confirmed that ratios of Pu­(IV) and Pu­(VI) concentrations in the samples did not change appreciably in this time frame.

### Multivariate Data Analysis

#### Spectral Preprocessing

Chemometric modeling was used to develop an understanding of the individual effects of HNO_3_ concentration and temperature on Pu­(IV) vis–NIR spectral features. The raw spectra were baseline corrected using asymmetric least-squares^23^ with smoothness parameter λ = 1 × 10^5^ and asymmetry parameter *p* = 0.01. The parameters were optimized by visual inspection of corrected spectra. Additionally, for PCA and PLSR analysis, the 820–840 nm region was excluded because this region contains variation due to the Pu­(VI) peak at 831 nm, which occurs for samples with low HNO_3_ concentration. Data analysis was performed in R version 4.5.2 (R Foundation for Statistical Computing, Vienna, Austria), with partial least-squares (PLS) and PCA evaluated using the *mdatools* package.^24^

#### Principal Component Analysis

PCA models were built to give an exploratory assessment of the patterns in the samples related to HNO_3_ concentration and temperature variation. An initial model was built using all acid concentration levels (0.6–9.5 M) to display global trends, and three smaller PCA models were then built on subsets of the HNO_3_ levels to reveal more localized trends: low (0.6–1.3 M), intermediate (2.3–6.0 M), and high (7.0–9.5 M) acid concentration. Loadings analysis was evaluated to give an approximate assessment of the most influential variables, with particular emphasis on those involved in temperature and HNO_3_ variation.

#### PLSR Models

PLSR is the most commonly used regression method in chemometrics.^25^ For a data matrix **X** of *m* samples and *n* variables and a property vector **y** of length *m*, PLSR will calculate a set of latent variables that simultaneously explain the variation in **X** and the covariance between **X** and **y**. Chemometric models for online monitoring of radioisotope processing are typically built using lab-scale data that are collected without varying sample temperature.^12,26^ In practice, such models would be deployed to predict features of in situ processing data in production environments that may vary significantly in temperature, which affects the spectral features of Pu­(IV). Several different validation strategies were assessed for incorporating temperature variation in the prediction of HNO_3_ concentration using PLSR.

The data were split into a training set consisting of eight different acid levels (0.6, 1.3, 2.3, 3.3, 5, 6, 7, and 9.5 M HNO_3_) and a test set with three acid concentration levels (1, 4.3, and 8 M HNO_3_). The levels for the test set were selected such that the prediction performance would be evaluated on low, medium, and high acid levels in order to test the ability of the model to predict data at HNO_3_ concentrations not included in the training set. The levels of 1, 4.3, and 8 M HNO_3_ were chosen so that low, medium, and high HNO_3_ concentrations, respectively, were represented in the test set. The number of PLSR components was optimized using an 8-fold cross validation on the training set. For each fold, one of the acid concentration levels was used as the holdout set. The test set samples featured all temperatures (15, 20, 30, 35, 40, and 45 °C) at the three acid levels (1, 4.3, and 8 M HNO_3_), for a total of 18 samples.

Three training sets were constructed to assess different assumptions about calibration temperatures used in PLS models. The first training set, called low-temperature, consisted of 16 samples at laboratory temperatures (15 and 20 °C). The second training set, three-temperature, consisted of 24 samples with the lowest, highest, and an intermediate temperature (15, 30, and 45 °C). The third training set, all-temperature, used 48 training and test set samples that spanned the range of 15–45 °C.

The prediction performance was assessed using the root-mean-square error (RMSE) metrics corresponding to predictions on the calibration set (RMSEC), cross validation (RMSECV), and test set predictions (RMSEP). The RMSE is defined as

$$\mathrm{RMSE}=\sqrt{\frac{1}{n}\sum_{i=1}^{n}({\hat{y}}_{i}-y_{i}{)}^{2}}$$ 1

where *n* is the number of samples, *ŷ*_*i*_ is the predicted concentration, and *y*_*i*_ is the measured concentration. Additionally, the percent RMSE (%RMSE) is defined as

$$\%\mathrm{RMSE}=\frac{\mathrm{RMSEP}}{y_{\mathrm{avg}}}\times 100$$ 2

where *y*_avg_ is the average concentration over the set evaluated (training/cross validation/test).

## Results and Discussion

### Pu Absorbance Spectra

The combined effects of HNO_3_ concentration and temperature produce substantial changes in the characteristic peaks of the Pu­(IV) spectrum at approximately 476, 543, 659, and 802 nm.^7,8^ Spectral features of Pu­(IV) are known to depend upon HNO_3_ concentration due to the formation of multiple Pu­(IV) nitrato complexes in solution.^11–13^ Previous work indicates that the Pu­(IV) aquo complex is stable in dilute HNO_3_ and that as HNO_3_ concentration increases, the number of coordinating nitrate ligands also increases, which was noted for the analogous Np­(IV) system.^6,27–29^ Experimental data and thermodynamic modeling indicated the formation of mono-, di-, and tetranitrato Pu­(IV) complexes, and more than one species can possibly exist in solution.^30–34^ At HNO_3_ concentrations of 10 M and higher, spectroscopic data indicate that the Pu­(IV) hexanitrato complex is the dominant species.^29^ The speciation of Pu­(IV) in HNO_3_ is complex; however, the effect of temperature on Pu spectral features at different HNO_3_ concentrations is not well-studied.^1,14^

Multiple temperature-induced changes in Pu spectral features over the low HNO_3_ concentration range (0.6–2.3 M) are associated with Pu­(IV) and its oxidation to Pu­(VI). Previous work suggests that the mono- and dinitrato Pu­(IV) complexes are most prominent over this acid range.^12,30^ Most spectral features decrease in intensity with increasing temperature, such as the signals at 404, 424, 476, 547, and 662 nm, as shown in Figure 1. Additionally, some features, such as the peak at 476 nm, also redshift to higher wavelengths with temperature (Figure 1B). Most notably, the 830 nm peak associated with Pu­(VI) does not follow this trend and instead increases in intensity with temperature, which indicates Pu oxidation.^11,13,14^ The change in Pu­(VI) peak intensity is most drastic in the 0.6 M HNO_3_ spectrum (Figure 1C, red trace), although this trend is also present to a lesser extent in the spectra of the 1, 1.3 and 2.3 M samples (green, yellow, and red traces, respectively). Additionally, in the 0.6 M HNO_3_ sample, a minor Pu­(III) component at 600 nm increases in intensity with temperature. The formation of Pu­(III) at low acid concentrations could be due to the combined disproportionation of Pu­(IV) to Pu­(III) and Pu­(VI).^13,34,35^ Little change is visible in the signals at approximately 540, 650, and 800 nm, which agrees with the study from Lascola et al.^1^ It is important to note that the temperature-dependent spectra of samples were collected as soon as was possible after sample synthesis, typically a few days to 1 week. This was to ensure that the sample had sufficient equilibration time after synthesis but was not stored for a prolonged period of time that could affect the Pu oxidation state ratios. Heating and data collection for one sample typically lasted approximately 45 min.

**Figure 1:**
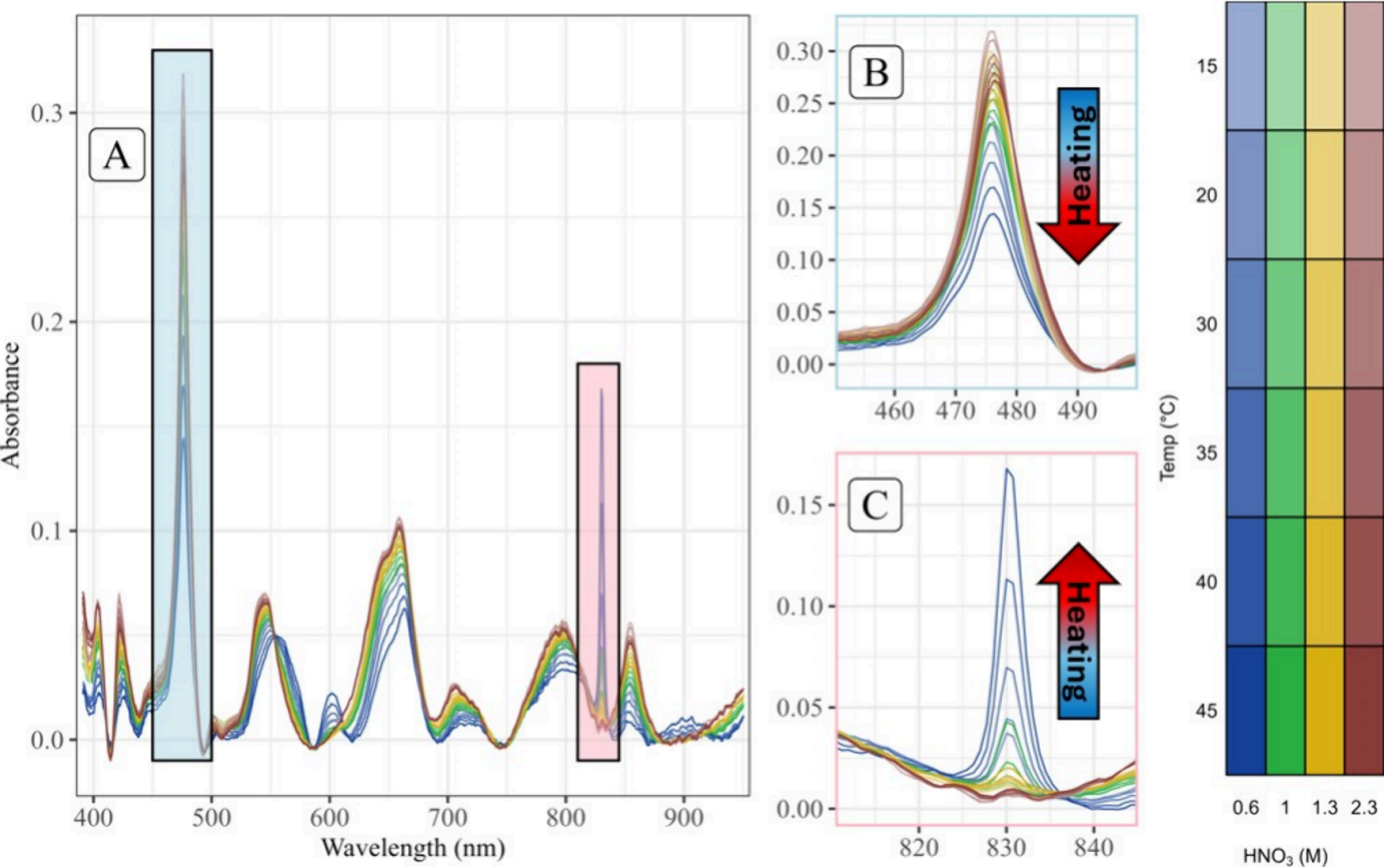
(A) UV–vis–NIR spectra of Pu­(IV) at low HNO_3_ concentrations: 0.6, 1, 1.3, and 2.3 M, (B) vis region of the spectrum between 450 and 500 nm, and (C) NIR region of the spectrum between 810 and 850 nm. Arrows in (B) and (C) indicate the direction of change in absorbance with increasing temperature.

In the intermediate acid concentration range from 3.3 to 5 M, temperature-induced changes in spectral features are more subtle than those shown in the low acid concentration range, with incremental changes in peak intensity and position. The di- and tetranitrato Pu­(IV) complexes are expected to dominate in this range, although contributions from the hexanitrato species are also feasible.^12,29^ Most signals decrease in intensity with increasing temperature, similar to the spectra for the low acid concentration system shown in Figure 2. However, a key difference between these two systems is that the intensity of the Pu­(VI) peak at 830 nm does not change significantly (less than 0.05 abs between the 15 and 45 °C spectra) with temperature for the samples in the intermediate acid concentration range (Figure 2C). Although the value of the molar extinction coefficient of Pu­(VI) is generally higher for lower HNO_3_ concentrations, spectral data suggest that the extent of oxidation to Pu­(VI) is not as prominent over this intermediate acid range.^11^

**Figure 2:**
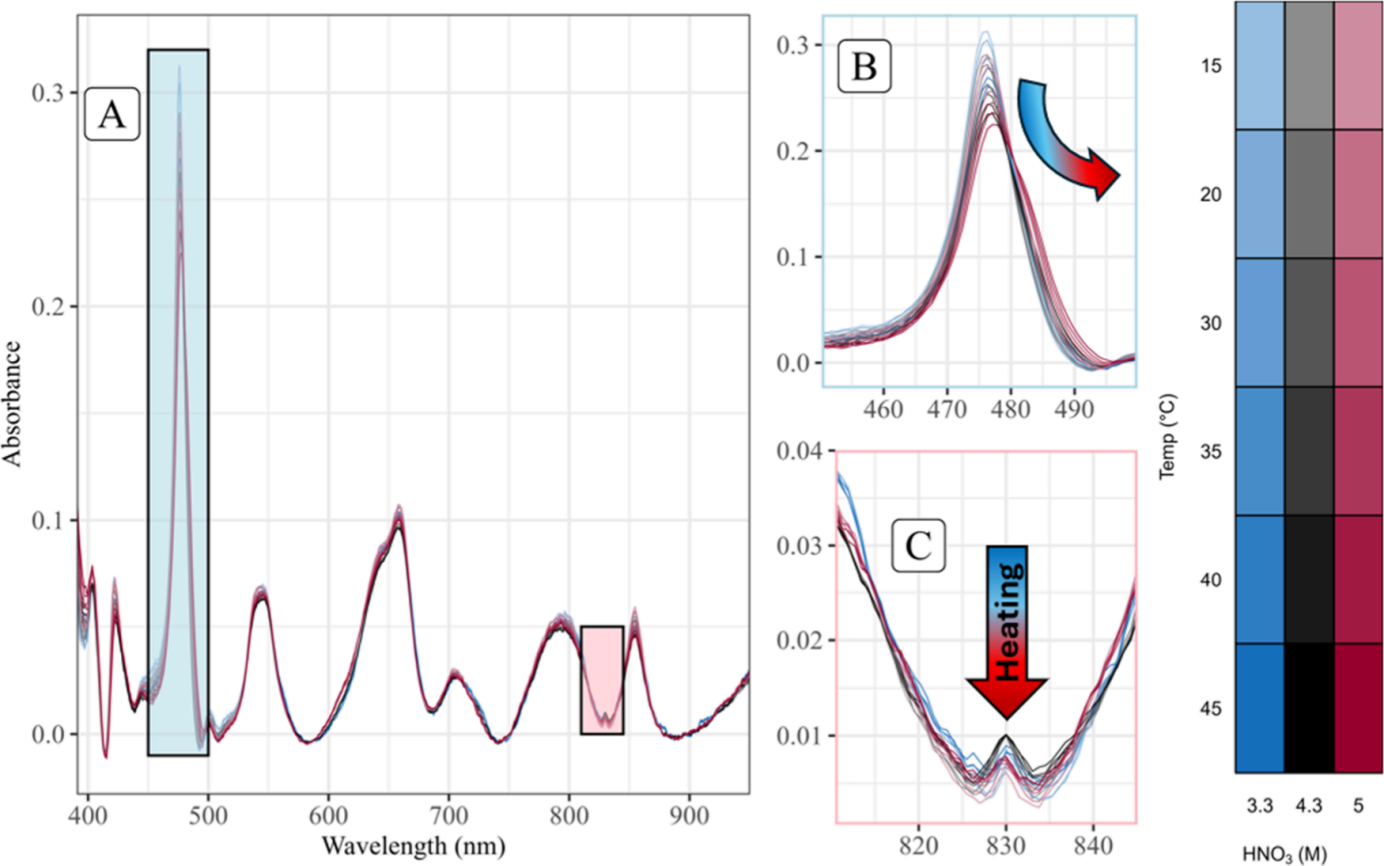
(A) UV–vis–NIR spectra of Pu­(IV) at intermediate HNO_3_ concentrations: 3.3, 4.3, and 5 M, (B) the vis region of the spectrum between 450 and 500 nm, and (C) NIR region of the spectrum between 810 and 850 nm. Arrows in (B) and (C) indicate the direction of change in absorbance with increasing temperature.

The 476 nm peak exhibits the most change in peak intensity and position with temperature (Figure 2B) for the samples in the intermediate acid concentration range. For all samples in the range, this peak systematically decreases in intensity and redshifts with increasing temperature. A shoulder also appears on the right side of the peak, which is most prominent in the 5 M HNO_3_ sample spectrum at 45 °C. The shape of the shoulder that appears with temperature is comparable with that of the shoulder that is visible in spectra of Pu at higher acid concentrations, such as 8 M HNO_3_. This could be due to changes in Pu­(IV) nitrato speciation; the formation constants of the Pu­(IV) nitrato complexes are known to depend on temperature.^15^

For the high acid concentration range from 6 to 9.5 M HNO_3_, changes in spectral features as a function of temperature are similar to those present in the intermediate acid concentration range. A mixture of Pu­(IV) nitrato species is expected over this acid range, with the tetra- and hexanitrato complexes most prominent.^12,29^ The spectrum of the 9.5 M HNO_3_ sample is notably different from those of the 6, 7, and 8 M HNO_3_, in agreement with the literature.^12,29^ These differences are attributed to the dominance of the hexanitrato complex at this acidity.^12^ Similar to the low and intermediate acid concentration ranges, most spectral bands decrease in intensity with increasing temperature, as shown in Figure 3. The signal centered at approximately 476 nm changes drastically with temperature when compared with changes observed in the corresponding signal over the low and intermediate acid concentration ranges. A significant redshift and broadening of this peak is shown as a function of temperature, with changes most notable in the 8 M HNO_3_ spectrum. The 830 nm peak is visible in all samples in the high acid concentration range, as well; however, the change in intensity of this peak with increasing temperature is quite small and is comparable with that of the intermediate acid concentration range. As such, changes in spectral features of these samples in the high acid concentration range are likely due to changes in Pu­(IV) nitrato speciation as opposed to Pu redox behavior, as was observed for samples in the intermediate acid concentration range.^1,15^ Notably, for all sample compositions in the current study, the changes in peak intensity due to temperature are generally more subtle than those attributed to HNO_3_ concentration.

**Figure 3:**
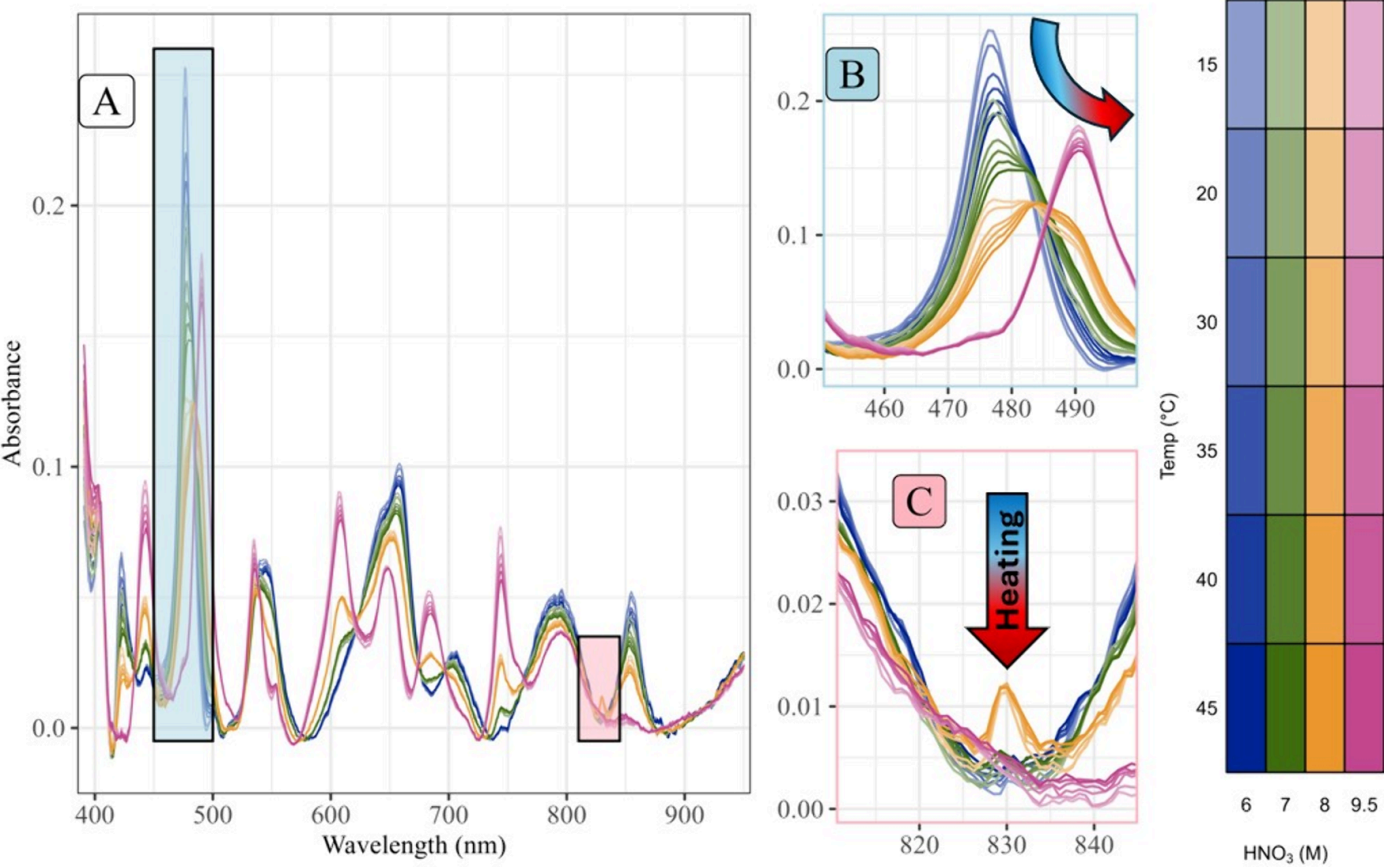
(A) UV–vis and (B) NIR spectra of Pu­(IV) at high HNO_3_ concentrations: 6, 7, 8, and 9.5 M, (B) vis region of the spectrum between 450 and 500 nm, and (C) NIR region of the spectrum between 810 and 850 nm. Arrows in (B) and (C) indicate the direction of change in absorbance with increasing temperature.

Differences in temperature-dependent changes that are observed in the spectral features of Pu samples over a range HNO_3_ concentrations suggest the influence of multiple mechanisms as opposed to a singular, uniform temperature effect. Oxidation of Pu­(IV) to Pu­(VI) and disproportionation of Pu­(IV) to Pu­(III) and Pu­(VI) appear to predominate particularly at low HNO_3_ concentrations. Other factors, such as temperature dependent changes in Pu nitrato speciation, spin–orbit coupling, and ligand field effects also likely contribute to changes in spectral features with temperature.^34,36,37^ Additionally, temperature is known to cause shifts in redox potentials and equilibrium constants for the Pu system.^38^ It is important to note that it is difficult to determine which effects contribute to changes in a singular feature in the absorbance spectrum.^37^ Previous work has underscored the importance of temperature in Pu chemical behavior, even on other oxidation states of Pu. For example, significant variation in the absorbance spectra of Pu­(VI) was reported due to temperature-driven hydrolysis reactions.^36^

### Multivariate Analyses of Pu­(IV) Absorbance Spectra

#### PCA for Temperature Effect on Spectra

The spectral data presented here provide a unique opportunity to evaluate the effects of HNO_3_ concentration and temperature on Pu­(IV) UV–vis–NIR spectra. To explore these major sources of variation, the baseline-corrected spectra were mean-centered, and a PCA model was built using the 390–820 and 840–950 nm regions of the spectra. The 820–840 nm region was excluded due to the presence of the 831 nm Pu­(VI) peak. The PCA scores plot for the first two components when using all samples in a global model is shown in Figure 4A, with corresponding loadings given in Figure 5A. For the global model, the first principal component (PC1), second principal component (PC2), and third principal component (PC3) explain 80.3, 14.3, and 4.3% of the variation, respectively. The contribution of additional PCs to explained variance is given in the Supporting Information (SI) in Figure S2. The scores show a systematic pattern with an arc shape, and the dominant clustering is based on the HNO_3_ levels. Additionally, all HNO_3_ levels except the 9.5 M samples share a pattern in which the scores on PC1 increase and become more positive with increasing temperature. Along PC1, the positive loadings are at 391, 441, 490, 606, and 743 nm, and negative loadings are at 421, 476, and 662 nm. Along PC2, positive loadings are present for 391, 483, 537, 630, 656, and 855 nm, and a negative peak is present at 562 nm. Examination of the PCA scores plot for PC1 and PC3 (Figure S4) also reveal trends which indicate that PC3 shows a relationship to temperature variation for the global model. More discussion is given in the SI.

**Figure 4:**
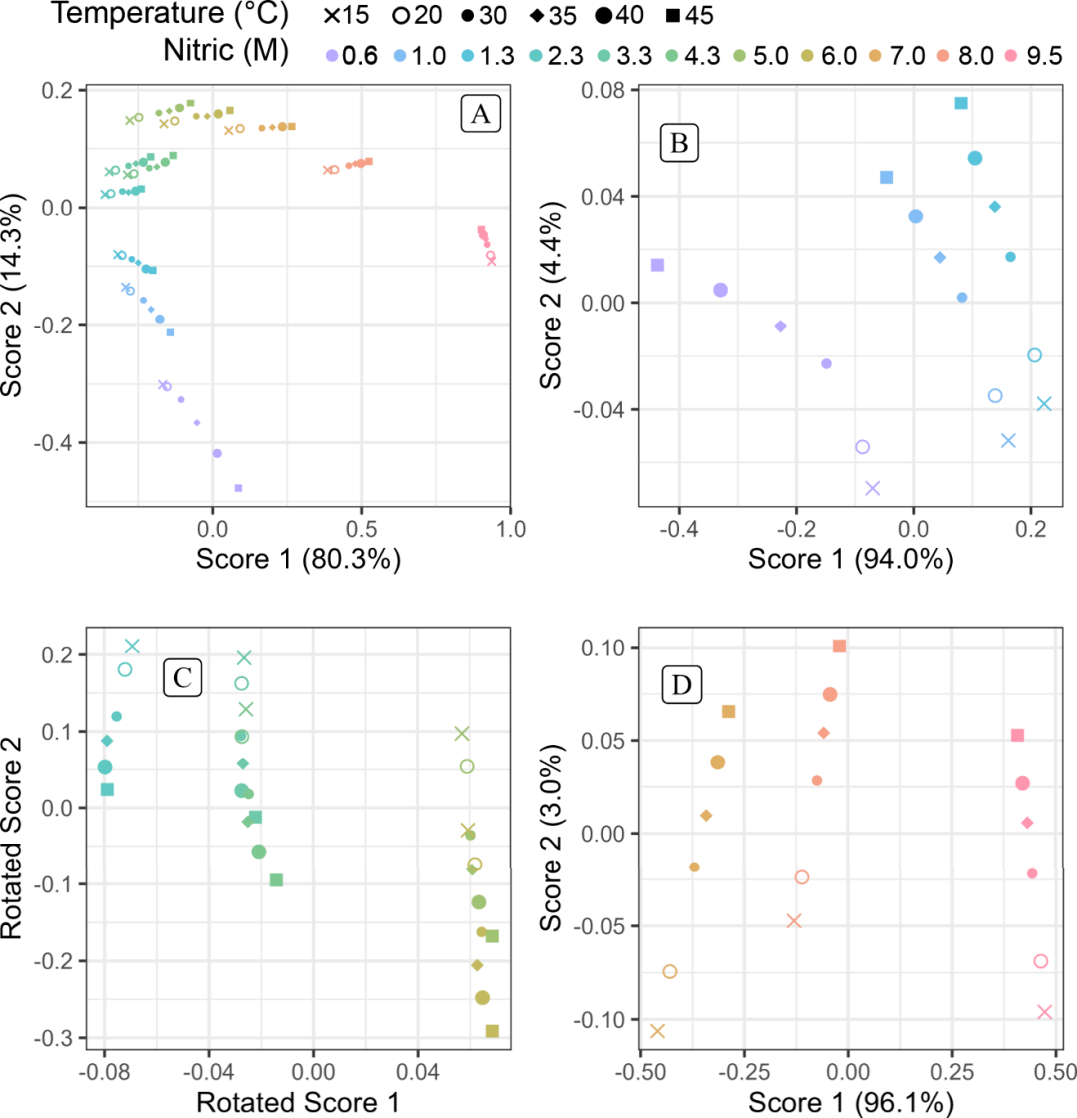
PCA scores plots of (A) global (0.6–9.5 M), (B) low acid concentration (0.6–1.3 M), (C) intermediate acid concentration (2.3–6.0 M), and (D) high acid concentration (7.0–9.5 M) models. Intermediate acid concentration (2.3–6.0 M) scores were rotated by 8π/5 (288°).

**Figure 5:**
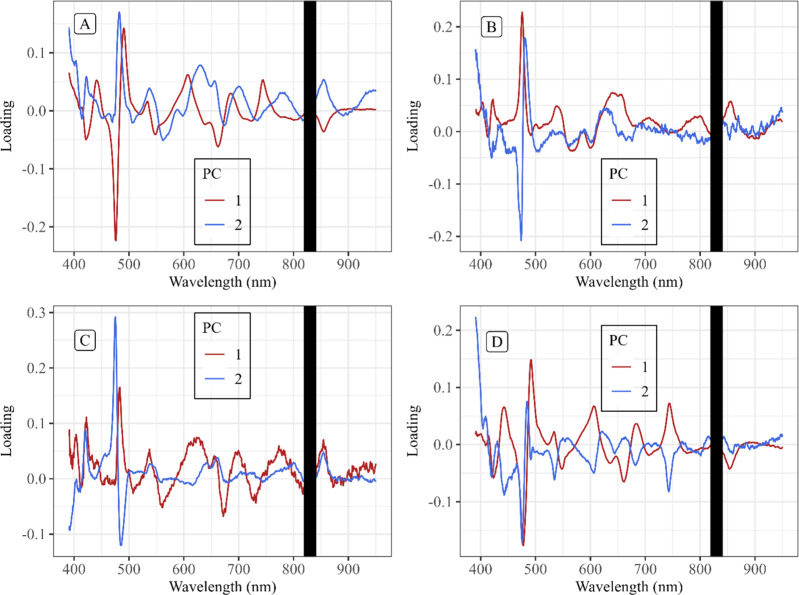
PCA loadings plots of (A) global, (B) low acid concentration, (C) intermediate acid concentration, and (D) high acid concentration models. Intermediate acid concentration loadings were rotated by 8π/5 (288°).

To further examine sources of variation in the spectra, PCA models were calculated on three smaller subsets of samples: low acid concentration levels (0.6–1.3 M HNO_3_), intermediate acid concentration levels (2.3–6 M HNO_3_), and high acid concentration levels (7–9.5 M HNO_3_). Samples were mean-centered relative to the mean of the subset.

The scores and loadings from the first two principal components (PCs) for the low-acid PCA model are shown in Figures 4B and 5B. These PCs describe 98.4% of variation in this data set. For each acid level, as temperature increases, the scores along PC1 are more negative and more positive along PC2. The 1 and 1.3 M samples are more similar to one another than to the 0.6 M samples, which aligns with the behavior in Figure 1. Along PC1, the largest loading is at 475 nm. Other positive peaks in the loadings are present at 403, 422, 537, 640, and 855 nm, and negative loading values are present at 564 and 599 nm. Along PC2, positive loadings are present at 391, 480, and 626 nm, and negative peaks are present at 419 and 473 nm. The loadings suggest that the signal at approximately 476 nm contributes to a significant amount of spectral variation across these acid levels and temperatures.

The scores and loadings from the first two PCs for the intermediate acid concentration PCA model are shown in Figures 4C and 5C, respectively. The scores plot shows some straight-line patterns of variation in temperature and acid concentration levels, but these patterns are a mixture of PC1 and PC2. To aid with interpretation, an operation called rotation was applied to the scores and loadings to rotate by an angle 8π/5 (288°).^39^ The transformation results in PC1 corresponding with variation due to acid concentration level, although the levels of 3.3 and 4.3 M, as well as 5 and 6 M, overlap strongly with one another. PC2 shows a strong negative correlation with the temperature levels. Along PC1, the loadings feature positive peaks at 391, 403, 422, 483, 623, and 855 nm, and negative peaks are at 561 and 672 nm. For PC2, the loadings are positive at 421 and 475 nm, and negative peaks are at 391 and 485 nm.

The scores and loadings from the first two PCs for the high acid concentration PCA model are shown in Figures 4D and 5D. PC1 primarily corresponds to acid concentration variation and accounts for 96.1% of variation, and PC2 shows a strong positive correlation with temperature. Along PC1, positive peaks in the loadings are present at 442, 491, 607, 683, and 744 nm, and negative loadings are present at 423, 478, and 661 nm. In PC2, the only prominent positive loadings peaks are at 391 and 484 nm, whereas negative loadings peaks are found at 420, 442, 475, 534, 607, and 743 nm. The findings of these PCA models underscore the nonuniform effect of temperature on Pu­(IV) absorbance spectra and that this effect depends on the HNO_3_ concentration of the Pu sample.

### PLS Models for Prediction of [H^+^] from Pu­(IV) Vis–NIR Spectra

As mentioned previously, temperatures of ambient laboratories and harsh radiological environments used for nuclear processing typically vary by multiple degrees Celsius. To assess the effect of temperature on model performance for HNO_3_ quantification, three PLSR models with calibration sets containing data collected at different temperatures were constructed. The low-temperature model used a calibration set with data collected at 15 and 20 °C, representative of laboratory conditions. The three-temperature model used a calibration set that included data collected at higher temperatures, specifically at 15, 30, and 45 °C. The all-temperature model used a calibration set with data collected at six different temperatures between 15 and 45 °C. Additional details of each model are given in Table 2.

**Table 2:** Summary of PLSR Models for the Prediction of HNO_3_ Concentration

**calibration set**	**NLV** ^a^	**RMSEC** (M, %)	**RMSECV** (M, %)	**RMSEP** (M, %)
low temperatures (15, 20 °C)	2	0.284 (11.2%)	0.49 (11.2%)	0.57 (12.8%)
three temperatures (15, 30, 45 °C)	3	0.41 (9.3%)	0.57 (13.1%)	0.39 (8.9%)
All temperatures (15, 20, 30, 35, 40, 45 °C)	4	0.23 (5.3%)	0.31 (7.1%)	0.23 (5.2%)

a Number of latent variables in PLS model.

The PLSR results are summarized in Table 2. Parity plots for each model, which compare the measured and predicted values from cross validation and test sets, are shown in Figure 6. For each model, the optimal number of latent variables was determined by assessing the percent RMSECV (%RMSECV) as a function of the latent variables included in the model (see Figure S5). The selected number of latent variables is two for the low-temperature model, three for the three-temperature model, and four for the all-temperature model. A variety of complex features are present in the data, such as overlapping peaks and changes in peak shape and position with HNO_3_ concentration and temperature. Consequently, multiple latent variables are required to adequately explain this behavior.

**Figure 6:**
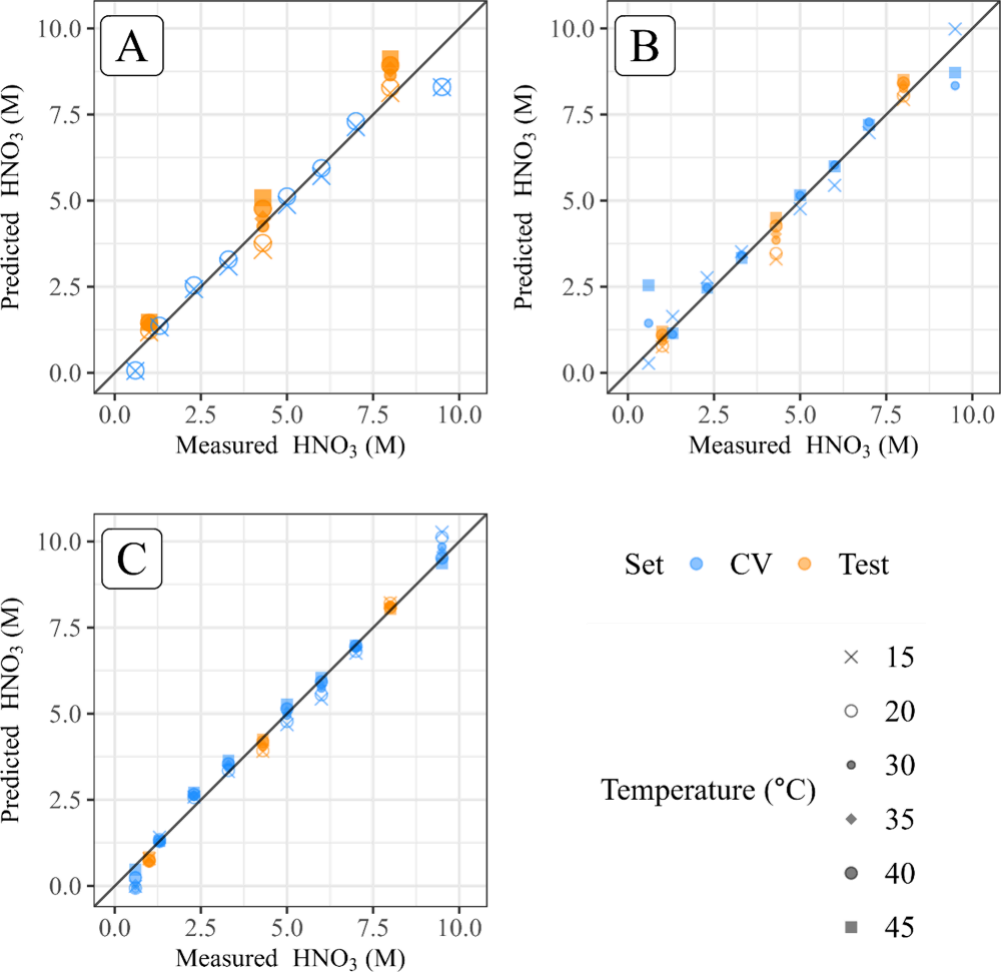
Parity plots for the three different PLS models: (A) low temperatures, (B) three temperatures, and (C) all temperatures.

The low-temperature model results in a lower RMSEC and RMSECV than the three-temperature model but has a higher RMSEP. The low-temperature model struggled to quantify HNO_3_ concentrations in data collected at higher temperatures than those of the calibration set, as indicated by the parity plot in Figure 6A. The lower RMSEP of the three-temperature model suggests that including data at higher temperatures in the calibration set improves the model’s predictive performance. The high RMSECV for the three-temperature model is attributed to the presence of large prediction errors for the 0.6 and 9.5 M samples, which are at the upper and lower edges of the calibration range, as shown in Figure 6B. However, the strongest model performance was observed in the all-temperature model, which has the lowest RMSEC, RMSECV, and RMSEP by a significant margin.

The PLSR coefficients were used to determine the importance of the different Pu­(IV) spectra features and are summarized in Figure S6. Coefficients between the three models are generally quite similar in the 450–500 nm region. The low-temperature coefficients are generally smaller in magnitude and feature a smoother profile, likely due to the lower number of components. The regression coefficients for the three-temperature model are generally intermediate compared with those of the low-temperature and all-temperature models. The coefficients for the all-temperature model show sharper peaks, including at 395, 421, 561, 609, 671, and 730 nm, which indicate that the model uses these particular features to predict acid concentration.

Overall, these results demonstrate the importance of accounting for temperature in calibration sets to optimize PLSR model performance. The low-temperature model displayed poor predictive performance and struggled to quantify HNO_3_ concentration of data collected at higher temperatures than those of the calibration set. By tuning the calibration set to include data collected at higher temperatures, as in the three-temperature model, predictive performance for HNO_3_ concentration improved by several percent. Finally, the strongest model performance was observed in the all-temperature model, which included data collected over a range of temperatures in the calibration set. These findings demonstrate that measuring and including Pu­(IV) absorbance spectra over a range of temperatures in calibration sets of PLSR models helps improve accuracy for HNO_3_ quantification and is crucial for strong model performance.

## Conclusions

This work constitutes the first systematic investigation of Pu­(IV) spectral features as a function temperature across a range of HNO_3_ concentrations. Changes in peak intensity, shape, and position were observed in spectra of all samples over the range of 0.6–9.5 M HNO_3_ with systematic heating of samples. The effect of temperature on Pu­(IV) absorbance spectral features appears to depend on HNO_3_ concentration. Variation in spectral features for samples in the low-concentration acid range is likely due to Pu­(IV) oxidizing to Pu­(VI), whereas at intermediate and high acid concentrations, spectral variation likely indicates changes in Pu­(IV) nitrate complexation.

Although the changes in Pu­(IV) absorption spectra with temperature appear subtle, PCA models of asymmetric least-squares baseline-corrected spectra reinforce that the contribution of temperature to spectral variation is nonuniform and depends on HNO_3_ concentration, especially in the primary peak at 476 nm.

Informed by findings from the PCA models, PLSR models were built using calibration sets of spectra collected at different temperatures to investigate the influence of temperature on model performance—specifically, HNO_3_ concentration prediction.

Model performance provided insight into the importance of accounting for temperature when building predictive models for online monitoring. The PLSR model built with the low-temperature calibration set, representative of data collected under typical laboratory conditions, did not perform well in predicting HNO_3_ concentration from data collected at higher temperatures. However, model performance improved when the calibration set was adjusted to include lower and upper temperature bounds and an intermediate temperature, as in the three-temperature model. Finally, model performance was optimized when the calibration set included data at all temperatures. These findings confirm the importance of accounting for temperature to build models for online monitoring that perform with strong predictive performance.^40^

This approach can be generalized to other systems to provide insight into the effect of process conditions on absorbance spectra of metal ions. In doing so, this work may help optimize the performance of chemometric models that are used to help online monitoring. Future work should continue to explore the temperature dependence of other actinide systems and expand on the wide range of feedback that online monitoring is capable of providing.

## Supplementary Material



## Notes

Figures from PCA and PLSR models including explained variance, scores, and regression coefficients plots, and a representative sample heating profile (PDF)

## Notes

This manuscript has been authored by UT-Battelle, LLC, under contract DE-AC05-00OR22725 with the US Department of Energy (DOE). The US government retains and the publisher, by accepting the article for publication, acknowledges that the US government retains a nonexclusive, paid-up, irrevocable, worldwide license to publish or reproduce the published form of this manuscript, or allow others to do so, for US government purposes. DOE will provide public access to these results of federally sponsored research in accordance with the DOE Public Access Plan (https://www.energy.gov/doe-public-access-plan).

## Abbreviations

NIR: near-infrared
PC: principal component
PC1: first principal component
PC2: second principal component
PCA: principal component analysis
PLS: partial least-squares
PLSR: partial least-squares regression
RMSE: root-mean-square error
RMSEC: root-mean-square error of calibration
RMSECV: root-mean-square error of cross validation
RMSEP: root-mean-square error of prediction
UV: ultraviolet
Vis: visible
