# Supplementary material for: Quantifying Temperature Dependence of Pu(IV) Absorbance Spectra for Advanced Online Monitoring of Nuclear Processes
Source: Anal Chem. 2026 Jun 20;98(27):20296–305. doi: 10.1021/acs.analchem.6c01635 (PMC13373921; doi:10.1021/acs.analchem.6c01635)
Supplement: Supplementary file 1 [file ac6c01635_si_001.pdf]

Supporting Information for:

Quantifying Temperature Dependence of Pu(IV) Absorbance Spectra for Advanced Online Monitoring of Nuclear Processes

Sara E. Gilson, Cannon J. Giglio, Hunter B. Andrews, Kristian G. Myhre, Luke R. Sadergaski*

Radioisotope Science and Technology Division, Oak Ridge National Laboratory, 1 Bethel Valley Road, Oak Ridge, TN 37830, USA

Table of Contents

Supplemental Graphs……..……………………………………………………S2

Experimental…………………………………………………………….S2

Principal Component Analysis (PCA)……………………………………..S3

Partial Least Squares Regression (PLSR)………………………………….S6

Supplemental Graphs

Experimental

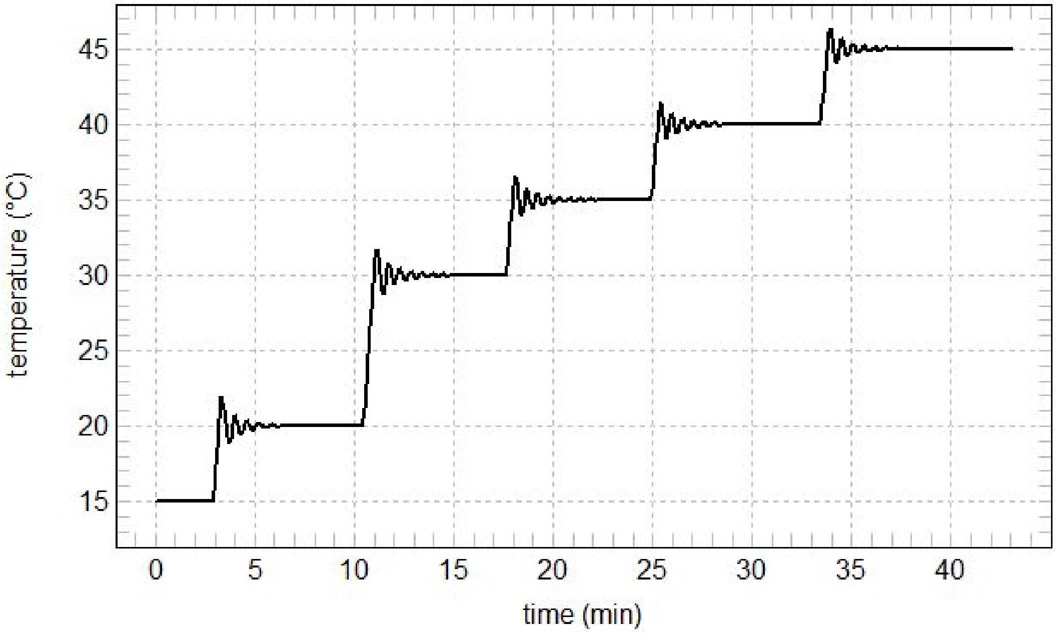

Figure S1. Representative heating protocol used for acquiring absorbance spectra.

Principal Component Analysis (PCA)

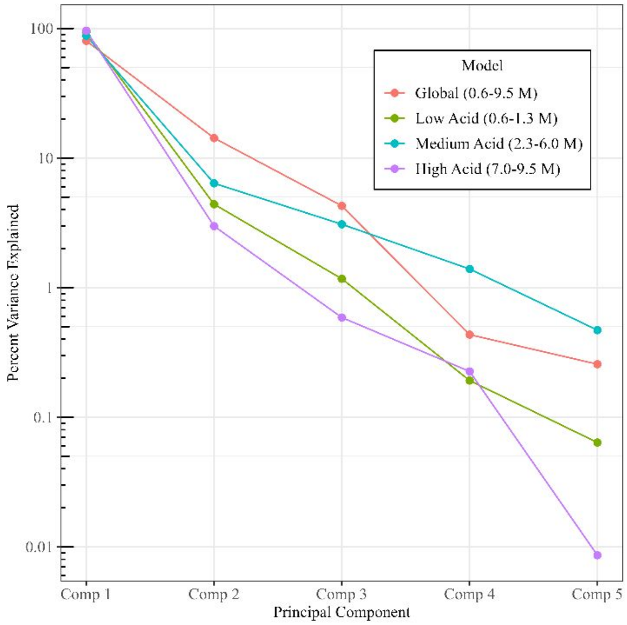

Figure S2. Percentage of variance explained versus number of components for global (0.6-9.5 M), low acid (0.6-1.3 M), intermediate acid (2.3-6.0 M), and high acid (7.0-9.5 M) PCA models. The y-axis is displayed using a logarithmic scale.

For the global model, the first three PCs are significant, accounting for 98.9% of variation in the baseline-corrected spectra. For the low and high acid models, the first two components are significant and account for over 98.4% and 99.1% of cumulative variance explained, respectively. For the medium acid PCA model, the third component explains 3.1% of variation and is moderately impactful. These differences are likely due in large part to the sample sizes, with the global model included 66 samples, the low and high acid models had only 18 samples, and the medium acid model used 30 samples.

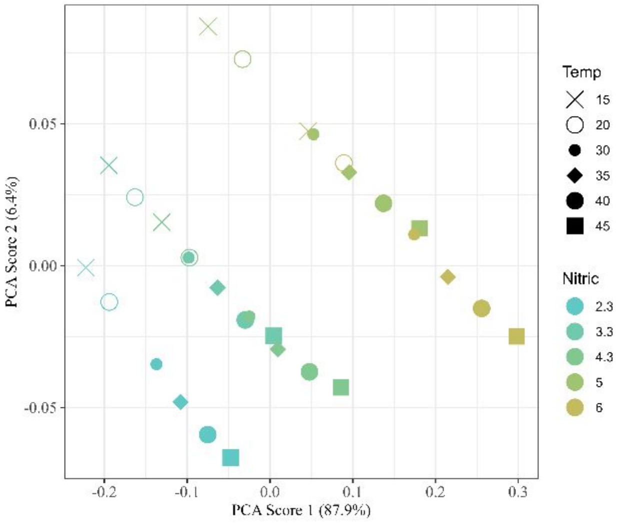

Figure S3. PCA scores for intermediate HNO_3 concentration levels.

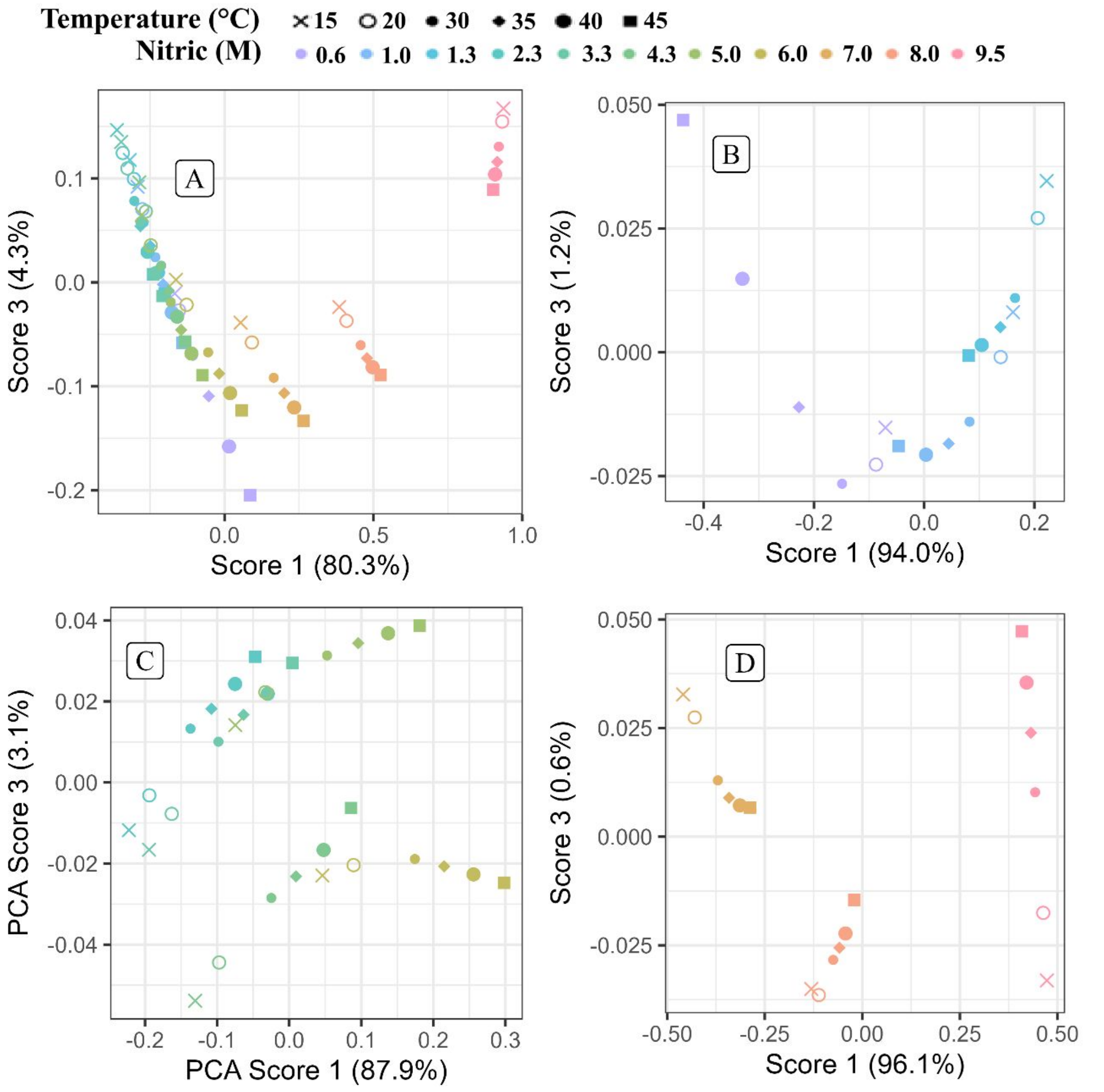

Figure S4. PCA scores plots of PC1 vs. PC3 for (A) global (0.6-9.5 M), (B) low acid (0.6-1.3 M), (C) intermediate acid (2.3-6.0 M), and (D) high acid (7.0-9.5 M) models.

For the global PCA model (Figure S4(A)), there is a consistent trend for each acid level along PC3, with low-temperature samples having more positive scores than high-temperature samples. In Figure S4(B) and S4(D), the scores do not show a consistent trend with respect to either acid or temperature. For the medium-temperature model, the 2.3-5.0 M samples have a consistent trend of increasing PC3 score with increasing temperature, but for the 6.0 M samples the PC3 scores hovered around -0.02.

Partial Least Squares Regression Analysis

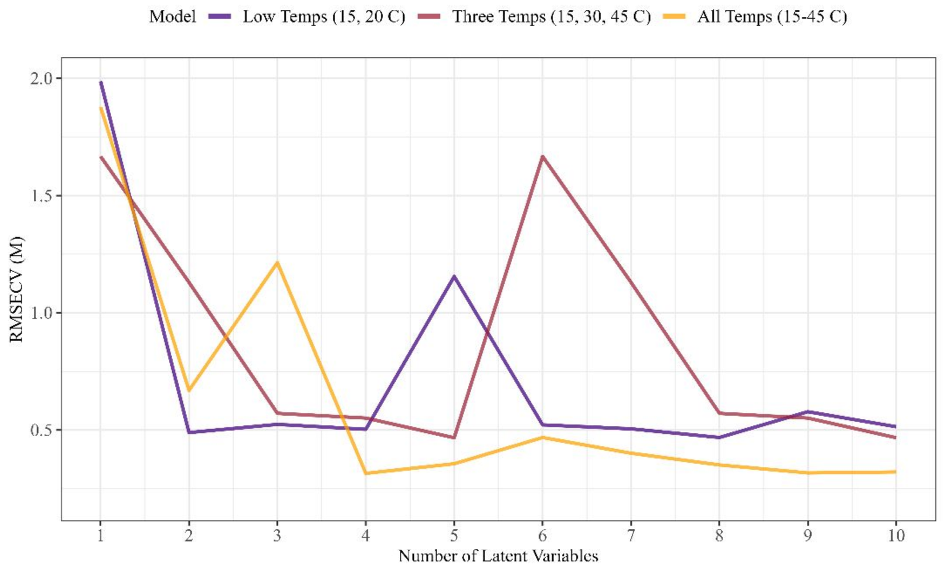

Figure S5. RMSECV vs. number of latent variables for each PLS model.

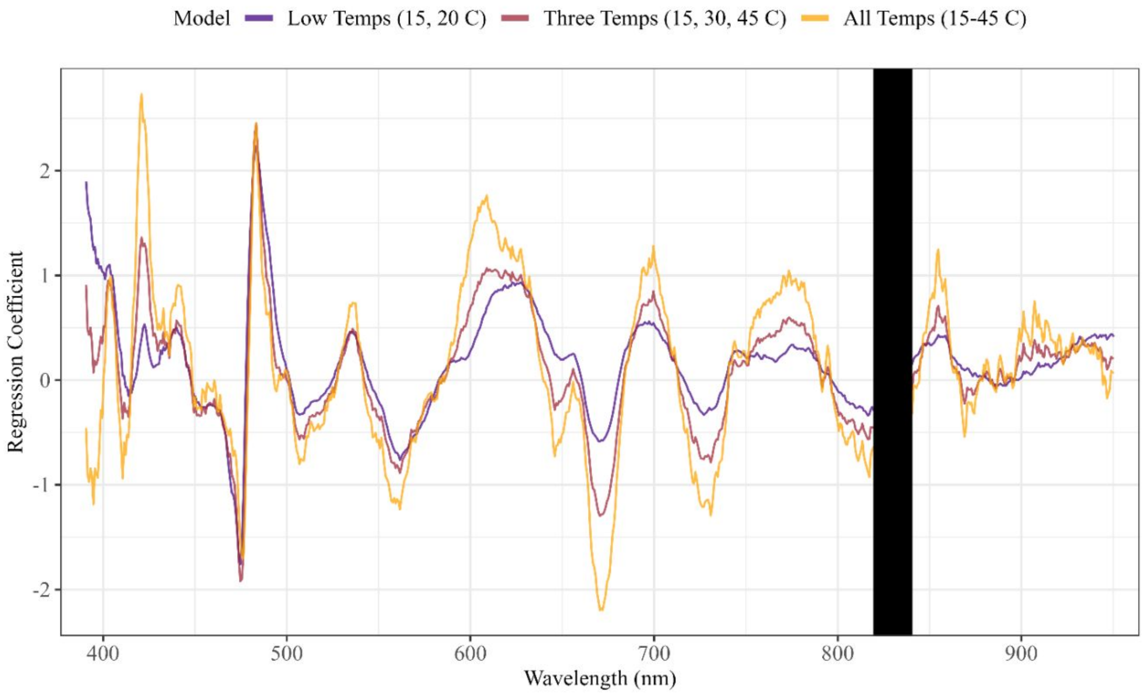

Figure S6. PLSR coefficients for the different models evaluated. The black vertical line indicates the 820–840 nm region, which was excluded from the models due to the presence of Pu(VI).
